# An Agreeable Cathartic

**Published:** 1876-02

**Authors:** 


An Agreeable Cathartic (*Berliner Klin. Wochenschrift* No. 41, 1875).—Dr. Paul Reich has been using during the past year a "tinctura rhamni frangulæ" (buckthorn), which he finds an agreeable form of administration of the cathartic, and one that is particularly appropriate to cases in which it has to be taken for a prolonged period. The usual dose of his tincture is two teaspoonfuls at night, but in some cases one suffices, causing a pultaceous stool the following morning. If a larger quantity is needed, he gives as much as four teaspoonfuls; if this does not act, he desists from the administration of the drug.

